# Synthesis of Silver Nanoplates with the Assistance of Natural Polymer (Sodium Alginate) Under 0 °C

**DOI:** 10.3390/ma13173827

**Published:** 2020-08-30

**Authors:** Pengfei Yang, Yu Liang, Daxiao Zhang, Jin Zhang, Shijie Li, Weiguo Liu

**Affiliations:** 1 Shaanxi Province Key Laboratory of Thin Films Technology and Optical Test, Xi’an Technological University, Xi’an 710032, China; pfyang@xatu.edu.cn (P.Y.); liangyu@st.xatu.edu.cn (Y.L.); j.zhang@xatu.edu.cn (J.Z.); lishijie@xatu.edu.cn (S.L.); 2 School of Physics and Technology, Wuhan University, Wuhan 430072, China; daxiao_zhang@whu.edu.cn

**Keywords:** silver nanoplate synthesis, sodium alginate, high repeatability, 0 °C

## Abstract

Some special conditions are important for chemical syntheses, such as high temperature and the medium used; unfortunately, uncontrollable influences are introduced during the process, resulting in unexpectedly low repeatability. Herein, we report a facile, environmentally friendly, stable, and repeatable methodology for synthesizing silver nanoplates (SNPs) at 0 °C that overcomes these issues and dramatically increases the yield. This method mainly employs sodium dodecyl sulfate (SDS) and sodium alginate (SA) as the surface stabilizer and assistant, respectively. Consequently, we produced hexagonal nanoplates and tailed nanoplates, and the characterization showed that SA dominates the clear and regular profiles of nanoplates at 0 °C. The tailed nanoplates, over time, showed the growth of heads and the dissolving of tails, and inclined to the nanoplates without tails. The synthesis method for SNPs used in this study—0 °C without media—showed high repeatability. We confirmed that these special conditions are not required for the synthesis of silver nanostructures (SNSs). Furthermore, we constructed a new method for preparing noble metal nanostructures and proved the possibility of preparing metal nanostructures at 0 °C.

## 1. Introduction

The optical, electrical, magnetic, biological, and catalytic properties of silver nanostructures (SNSs) are closely related to their shape and size [1,2,3,4,5]. Many kinds of SNSs with different morphologies, such as spheres, rods, wires, sheets, cubes, polyhedrons, branches, and so on [6,7,8,9,10,11,12], can be obtained using various methods. Compared with the defects produced by physical etching, as well as the limitations of microwave ultrasonic synthesis, chemical reduction is the most efficient and the least defective method for synthesizing SNSs [13,14,15,16].

Currently, many valuable SNSs have been produced via chemical reduction synthesis [17,18,19]. The reactions based on high temperature (>100 °C) are most popular; the high temperature during the reaction process and the poorly controllable accuracy affect the ability to synthesize the desired size of SNSs [20,21,22]. In contrast, the reactions working at low temperature (25–100 °C) are milder, and a strong reductant, such as electrodes or sodium borohydride, is usually used as a reducing agent to improve the reaction [23,24,25]. Medium seeds are also used to control the growth trend of nanostructures [26,27,28,29], which are driven by the greater number of steps in the reaction processes. Similar to the uncontrollable size at high temperature, other unexpected factors always follow more steps or media at low temperature. These reaction conditions, such as high temperature, medium seeds, strong reductants, and so on, appear to be required for the synthesis of SNSs, complicating the synthesis. In this paper, we show that the synthesis of silver nanoplates (SNPs) at 0 °C overcomes these issues. The factors negatively influencing the process are avoided as much as possible during preparation, successfully proving that these conditions are unnecessary. This is a new method for the synthesis of SNSs and the application of natural polymers.

During the synthesis of metal nanostructures, a surface stabilizer is integral for growth as it helps prevent nanostructures from aggregating and accelerates a specific direction during the growth process. A common polymer (poly (vinyl pyrrolidone) (PVP)) and long-chain alkyl (cetyl trimethyl ammonium bromide, CTAB/cetyl trimethyl ammonium chloride (CTAC)) often used at a temperature not lower than room temperature (~25 °C), which is not the optimum surface stabilizer for synthesizing SNSs at lower temperature (<25 °C) or even 0 °C [30,31,32,33,34,35,36,37]. Hence, sodium dodecyl sulfate (SDS), with a high degradation degree, is our preferred candidate for surface stabilizer when using a natural polymer sodium alginate (SA) as an assistant to precisely control the size and shape of SNSs [38,39].

In this study, we constructed a facile, environmentally friendly, and repeatable method that can be used to synthesize SNPs with the assistance of SA at 0 °C, and SNPs of hundreds of nanometers can be obtained in two hours without medium seeds. We verified that SNSs can be prepared at 0 °C and provide a new method for the synthesis of SNSs without the previously necessary requirements.

## 2. Materials and Methods

### 2.1. Materials

Sodium alginate (SA, 98%) was purchased from Shanghai Yuanye Bio-Technology Co., Ltd. (Shanghai, China). Sodium dodecyl sulfate (SDS) and AgNO_3_ (99.8%) were purchased from Sinopharm Chemical Reagent Co., Ltd. (Shanghai, China). L-ascorbic acid (AA, 99%) was purchased from Sigma-Aldrich (Munich, Germany). Deionized water with a resistivity of 18.2 MΩ cm, obtained from a Direct-Q 5 UV water purification system, was used in all experiments. All chemicals were used without further purification. The 0 °C temperature was controlled using a low-temperature thermostat (XDDC-2006, Shanghai Xida Instrument Co., Ltd., Shanghai, China).

### 2.2. Preparation

The 5 mM SDS aqueous solution was prepared by dissolving 144.2 mg SDS powder in 100 mL deionized water with stirring at 0 ± 0.02 °C for 20–30 min to ensure a homogeneous temperature. Then, SA (5 × 10^−4^ mg/mL; 10, 50, and 100 μL), AgNO_3_ (0.01 M, 0.4 mL), and AA (0.1 M, 50 μL) were added to the 15 mL SDS solution at a stable temperature in sequence and stirring was continued for 2 h. After the reaction, centrifugation (5000 rpm, 10 min, twice) was carried out within 30 min and the precipitates were cleaned with deionized water, then stored in an aqueous solution. For further characterization, natural air drying was used to obtain a certain quantity of SNPs. During the SNPs preparation process, a relatively low room temperature (16 ± 1 °C) was used to maintain the low fluctuation of the reacting temperature.

### 2.3. Characterization

Scanning electron microscope (SEM) images of samples were obtained using a Hitachi S-4800 (Tokyo, Japan) with an acceleration voltage of 5 kV. Transmission electron microscopy (TEM), high-resolution transmission electron microscopy (HRTEM) images, and energy-dispersive spectrum (EDS) of the SNPs were obtained using a JEM-2010 FEF transmission electron microscopes (JEOL Ltd., Tokyo, Japan) with an acceleration voltage of 200 kV. Fourier-transform infrared-attenuated total reflectance (FTIR-ATR) spectra of the samples was recorded using a Nicolet iS 5 FTIR spectrometer (Thermo Fisher Scientific Inc., Waltham, MA, USA) in the range of 400–4000 cm^−1^. The X-ray diffraction (XRD) patterns of dried nanoplates were recorded using a D8-Advance X-ray diffractometer (Bruker, Germany) in the 2θ range of 30°–80°.

## 3. Results

### 3.1. High-Yield Production of SNPs

**Figure 1:**
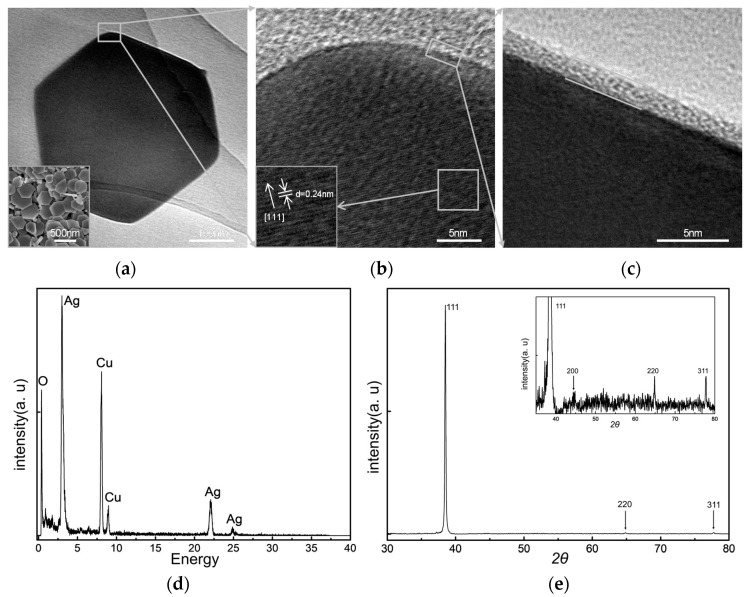
(**a**) The transmission electron microscopy image of silver nanoplate synthesized at 0 °C, with some tailed nanoplates shown in the insert of scanning electron microscope (SEM) image. (**b**) The high-resolution transmission electron microscopy (HRTEM) images show the lattice fringes and spacing of the silver nanoplate surface. (**c**) The HRTEM image shows the amorphous epitaxial layer on the edge of silver nanoplate. (**d**) The energy-dispersive spectrum of silver nanoplates (SNPs). (**e**) The X-ray diffraction spectrum of SNPs with four peaks, which correspond to the face-centered cube (fcc) silver crystal structures.

Using the proposed method of synthesizing metal nanostructures at 0 °C, we obtained regular hexagonal nanoplates with six sides of nearly equal lengths after reacting for two hours, as shown in Figure 1a. The results in the insert include most of the nanoplates and some tailed nanoplates, with clear and defined edge profiles, along with a small number of irregular particles. The lattice fringes of the nanoplates were observed under a HRTEM, and the fringe d = 0.24 nm in Figure 1b corresponds to {111} planes of silver, indicating the growth of the [111] plates. The amorphous epitaxial layer (AEL) on the edge in Figure 1c is the surface stabilizers surrounding the SNPs. The FTIR-ATR spectra of the SNPs in aqueous solution (see Supplementary Material, Figure S1) show the intense characteristic bands at 2920 and 1635 cm^−1^ corresponding to SDS (NaSO_4_C_12_H_25_) and SA (C_6_H_9_NaO_7_), respectively, and the band at 3454 cm^−1^ was mainly produced by an aqueous solution in the sample. It indicates that the surface-stabilizing layer of SNPs is the mixture of SDS and SA. The EDS of the nanoplates show one metal peak of Ag in Figure 1d (the peaks of Cu are caused by the copper grid), confirming that the nanoplates are silver and no other metal materials participate in the growth process. The EDS of the tails was similar to that of the nanoplates. The four characteristic XRD spectrum peaks are shown in Figure 1e and the insert image corresponds to the (111), (200), (220), and (311) of the face-centered cube (fcc) Ag crystal, which indicated the nanoplates were crystalline.

According to the characterizations of the samples, we speculated that at 0 °C, silver ions are initially reduced to form small silver nuclei with multiple crystal surfaces, providing a method for producing silver nanoseeds in situ [40]. As a surface stabilizer, SDS interacted with SA, enclosed the silver nanoparticles, and induced silver ions and atoms to accumulate primarily along [111], which is the reason why the (111) peak of SNPs shows the highest intensity in XRD patterns [41,42]. They extended outward at the side of the long-chain polymers supersaturated aggregation, which in turn reduced the silver ions, forming the tail as a result during this process.

### 3.2. Time Controlling the Growth of SNPs

**Figure 2:**
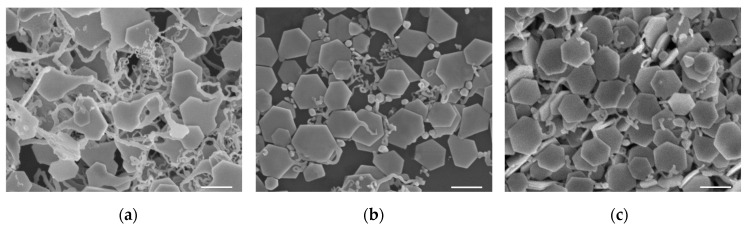
The SEM images of SNPs production under the same reaction conditions using 50 μL sodium alginate (SA) during synthesis with different reaction durations: (**a**) 1, (**b**) 1.5, and (**c**) 2 h. The scale bar is 500 nm.

For the growth of SNPs under the same synthesizing conditions, the time dependence of the samples is shown in Figure 2. The sample with a non-uniform size in Figure 2a is the product after reacting for one hour, showing rough edges and disorganized long tails following the nanoplates. Clear nanoplate edges appeared within 1.5 h, as shown in Figure 2b, and the nanoplates without tails gradually appeared. The hexagonal nanoplates in Figure 2c, produced with high yield and clear profiles, were obtained after two hours with a few with short tails, showing a more uniform size than the sample in Figure 2a. The hexagonal and tailed nanoplates (Figure S2), were obtained after growing 3, 4, and 5 h, showing clear profiles and the increasing size over time. The size and thickness distributions of the SNPs after growing 2 h are shown in Figure S3.

**Figure 3:**
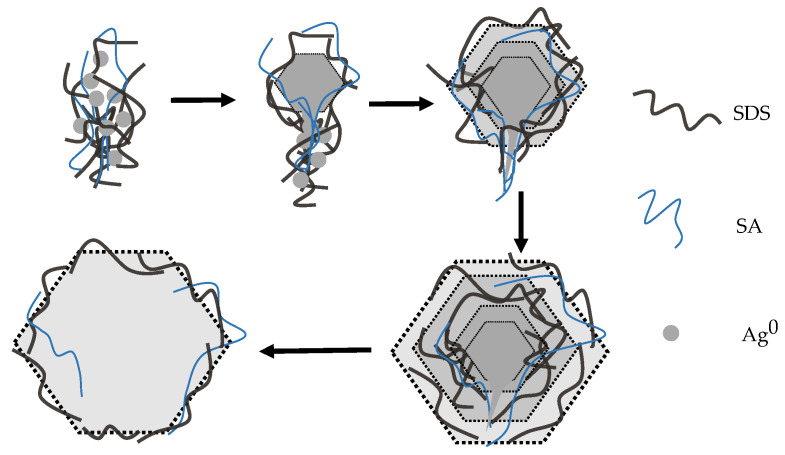
Schematic of the growth of nanoplates during the synthesis of SNPs under 0 °C.

The growth of SNPs over time involves the growing of plates and the dissolving of tails, showing a tendency for tails to disappear with time, which is the result of the chemical reaction. The tails and the plates initially have different growth rates during the synthesis process; subsequently, the nanoplates grow over time and parts of the tails are covered. The ends of tails formed by the supersaturated aggregation of polymers participating in the reduction of silver ions through the process of accumulating silver ions and consuming polymers; the parts of tails formed by silver atoms and nanoparticles will keep following the nanoplates as tails. Over time, both reactions occur simultaneously in the synthesis, producing the hexagonal and tailed nanoplates. Figure 3 shows the growth of nanoplates during the synthesis of SNPs under 0 °C and the tendency of the tails to disappear.

### 3.3. Morphological Changes in SNPs with Different Amounts of SA

**Figure 4:**
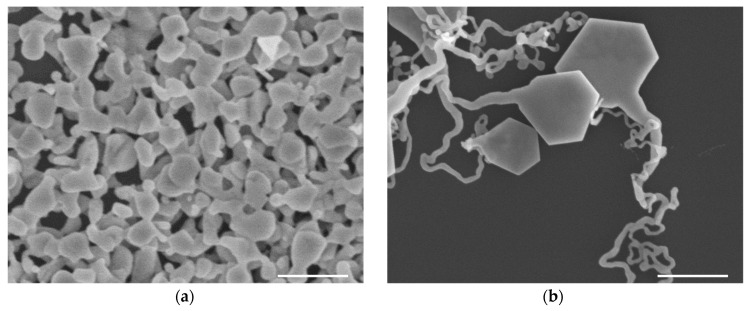

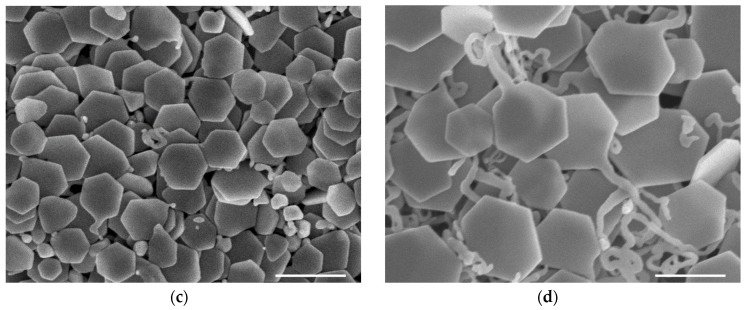
The SEM images about the results of using different amounts of SA in the synthesis of SNPs after 2 h: (**a**) blank experiment without SA; (**b**) 10, (**c**) 50, and (**d**) 100 μL SA. The scale bar is 500 nm.

To research the effect of the introduction of natural polymers for the synthesis of SNPs at 0 °C, we adjusted the amount of SA, and the morphology of the products changed dramatically. The results of the blank experiment (Figure 4a) showed irregular nanoplates with few corners and a relatively smooth local boundary, most of which were bonded with each other. Figure 4b depicts the sample produced by adding a small amount (10 μL) of SA. Clear edges and corners began to appear on the heads of the tailed nanoplates, while the tails were slender and twisted and extended into ultrathin lines. The products produced using 50 μL SA (Figure 4c) had regular hexagonal plates with six sides of nearly equal lengths and tailed plates with clear and defined edges. The tailed nanoplates in Figure 4d were obtained using 100 μL SA, which showed a tendency toward tail thinning and a silver particle at the end. These various shapes were the result of different amounts of SA introduced to the reaction, indicating that SA, as a natural polymer material, has an important influence on the synthesis of SNPs at 0 °C.

Compared with the results of the blank experiment without SA addition, we found that SA produced clear edges and the growth of tails. The SNPs with a little SA were controlled by SDS rather than fine control, so the continuous adhesion of silver ions and small particles led to an ultra-long and increasingly thin tail [43]. Similarly, during the reaction of excess SA compared with SDS, the silver ions and nanoparticles would accumulate along the SA due to the stronger induction and control for silver nanoparticles, which result in the tails maintaining stable radial growth and forming obvious irregular silver particles at the ends. Therefore, regular hexagonal nanoplates can only be produced using the optimum proportion of SA and SDS, with some tailed nanoplates also being produced during the synthesis. The results of two additional experiments that occurred under the same conditions as the experiment using 50 μL SA, as shown in Figures S4 and S5, showed hexagonal and tailed nanoplates similar with the sample in Figure 4c. This finding indicated that the method of synthesizing SNPs at 0 °C possesses high repeatability.

## 4. Conclusions

In this study, we synthesized hexagonal nanoplates at 0 °C without medium seeds, using SDS as the surface stabilizer and SA as the assistant, offering an environmentally friendly, stable, and repeatable synthesis route. The characterization and analysis of the experimental results showed that SA dominates the clear edges and assists the nanoplates to form uniformly over time when employing SDS as a surface stabilizer for the synthesis of SNPs at 0 °C. By obtaining the product under these conditions, we confirmed that high temperature and a medium are unnecessary for the synthesis of SNSs. We constructed a repeatable method for synthesizing noble metal nanostructures as well as a new research idea for the application of natural polymers. We also proved the possibility of synthesizing metal nanostructures at 0 °C.

## Supplementary Materials

The following are available online at https://www.mdpi.com/1996-1944/13/17/3827/s1, Figure S1: The Fourier-transform infrared-attenuated total reflectance spectra of the silver nanoplates (SNPs) stored in aqueous solution, Figure S2: The scanning electron microscope (SEM) images of SNPs produced under the same reaction conditions using 50 μL sodium alginate (SA) during synthesis with different reaction durations: (a) 3, (b) 4, and (c) 5 h. The scale bar is 500 nm, Figure S3: The size (S) and thickness (T) distributions of the SNPs after grown for 2 h in 50 μL SA, Figure S4: The SEM images of SNPs obtained after growing 2 h in the one of the two repeated experiments using 50 μL SA at 0 °C, Figure S5: SEM images of SNPs obtained after growing for 2 h in the one of the two repeated experiments using 50 μL SA at 0 °C.
