# Supplementary material for: Synthesis of Silver Nanoplates with the Assistance of Natural Polymer (Sodium Alginate) Under 0 °C
Source: Materials (Basel). 2020 Aug 30;13(17):3827. doi: 10.3390/ma13173827 (PMC7503834; doi:10.3390/ma13173827)
Supplement: Supplementary file 1 [file materials-13-03827-s001.pdf]

materials

MDPI

Supplementary Material

Synthesis of Silver Nanoplates with the Assistance of Natural Polymer (Sodium Alginate) Under 0 °C

Pengfei Yang [1,†], Yu Liang [1,†], Daxiao Zhang [2], Jin Zhang [1], Shijie Li [1] and Weiguo Liu [1,*]

[1] Shaanxi Province Key Laboratory of Thin Films Technology and Optical Test, Xi'an Technological University, Xi'an 710032, China; pfyang@xatu.edu.cn (P.Y.); liangyu@st.xatu.edu.cn (Y.L.); j.zhang@xatu.edu.cn (J.Z.); lishijie@xatu.edu.cn (S.L.)

[2] School of Physics and Technology, Center for Nanoscience and Nanotechnology, and Key Laboratory of Artificial Micro and Nanostructures of Ministry of Education, Wuhan University, Wuhan 430072, China; daxiao_zhang@whu.edu.cn

* Correspondence: wgliu@163.com; Tel.: +86-029-8320-8114

† These authors contributed equally to this work.

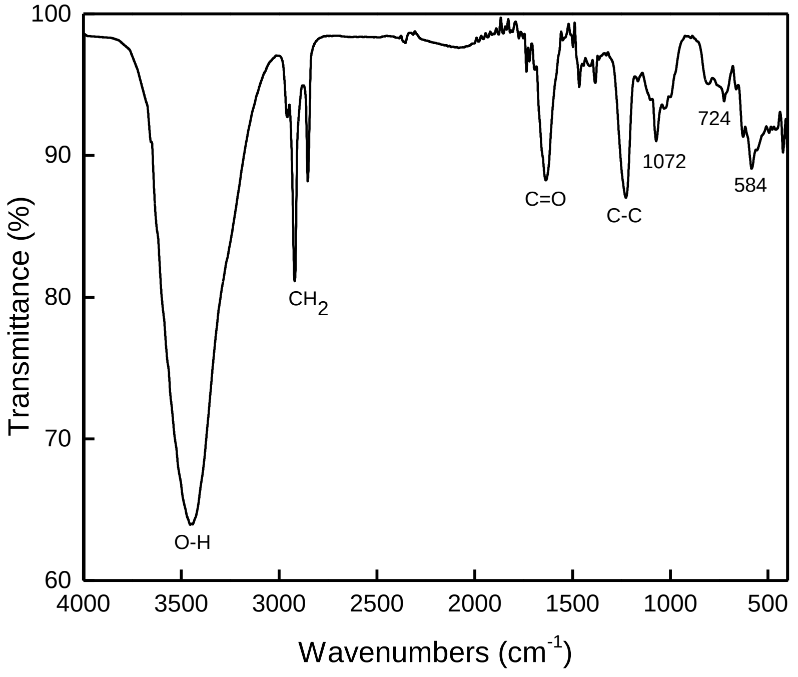

Figure S1. The Fourier-transform infrared-attenuated total reflectance spectra of the silver nanoplates (SNPs) stored in aqueous solution.

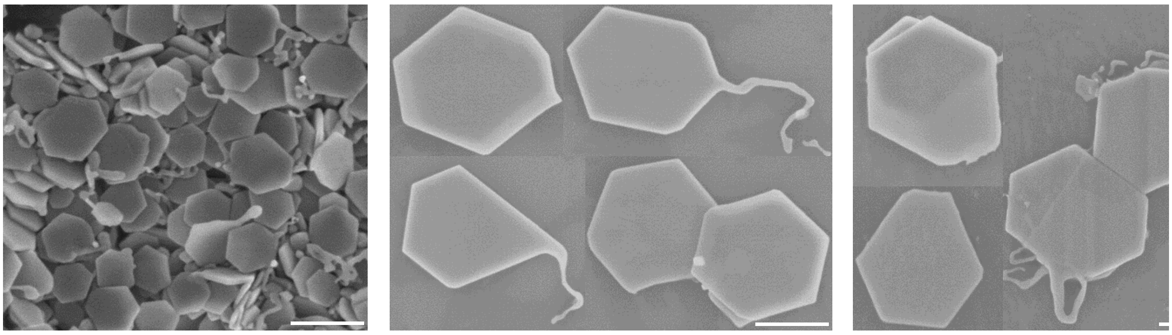

(**a**) (**b**) (**c**)

Figure S2. The scanning electron microscope (SEM) images of SNPs produced under the same reaction conditions using 50 μL sodium alginate (SA) during synthesis with different reaction durations: (**a**) 3, (**b**) 4, and (**c**) 5 h. The scale bar is 500 nm.

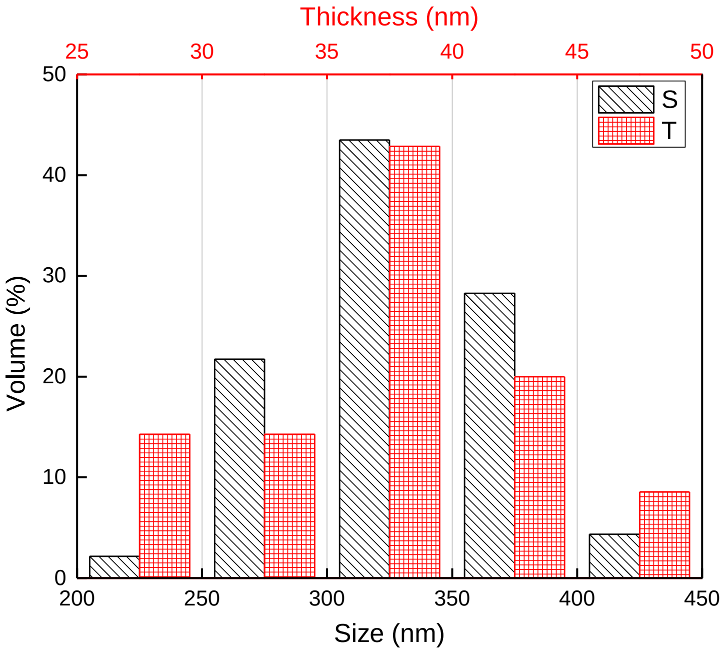

Figure S3. The size (S) and thickness (T) distributions of the SNPs after grown for 2 h in 50 µL SA.

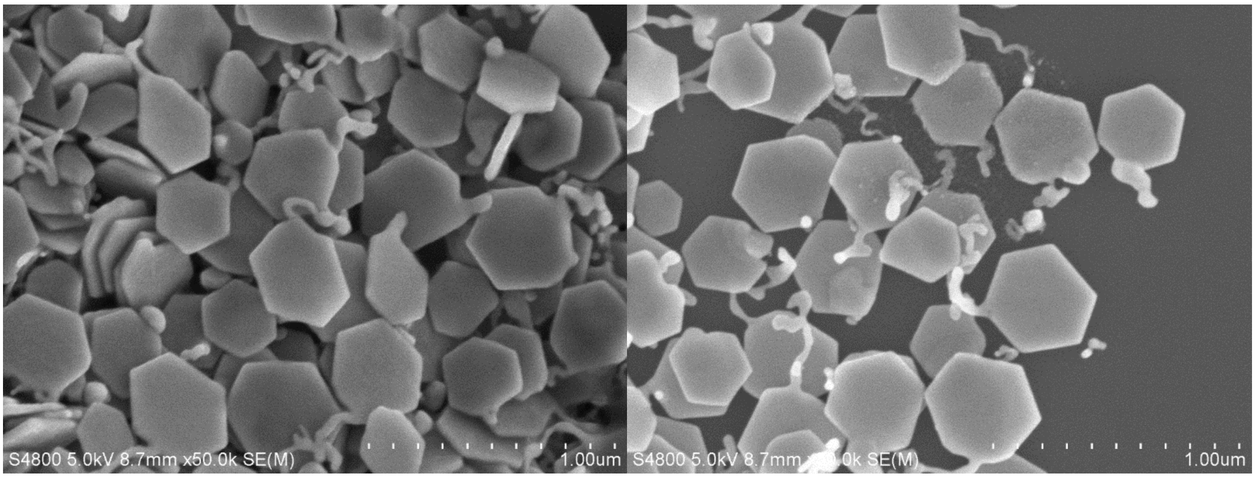

Figure S4. The SEM images of SNPs obtained after growing 2 h in the one of the two repeated experiments using 50 µL SA at 0 °C.

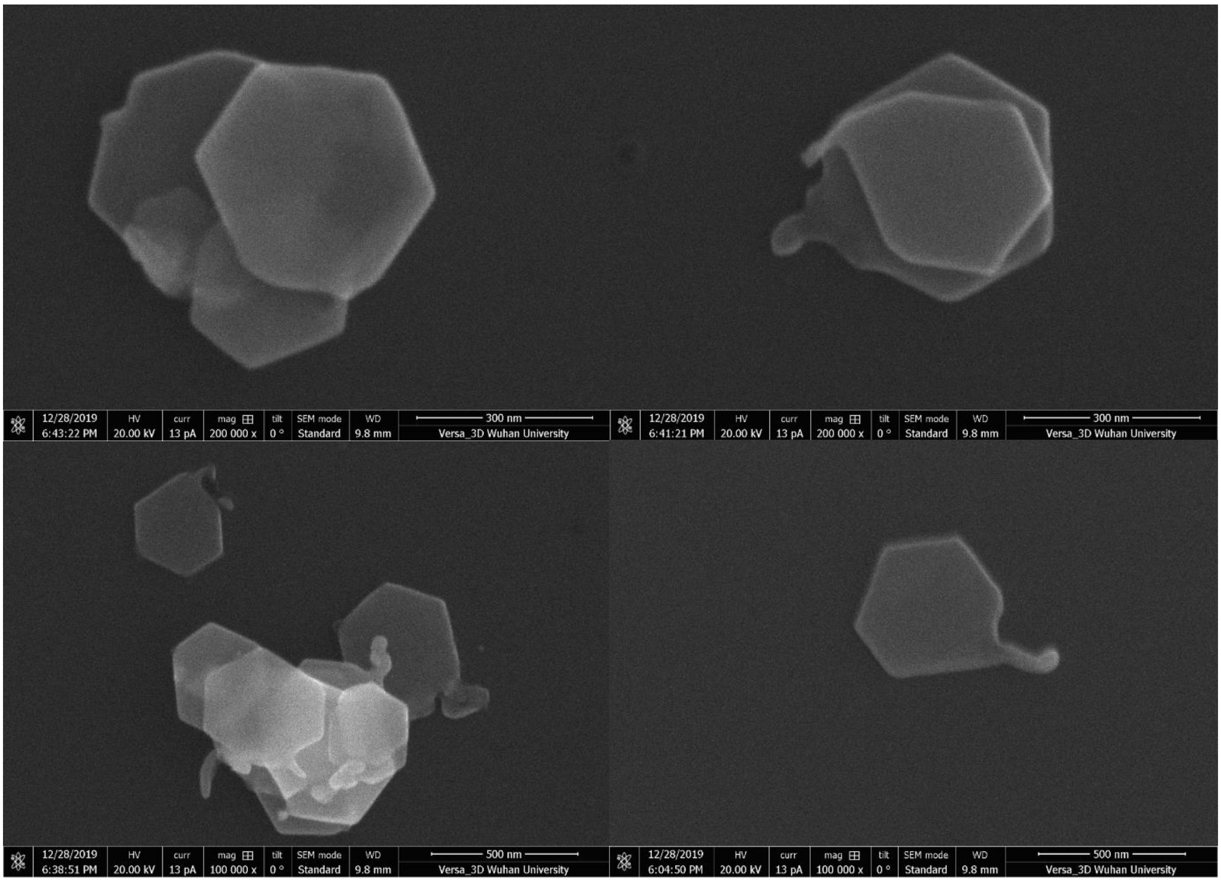

Figure S5. SEM images of SNPs obtained after growing for 2 h in the one of the two repeated experiments using 50 μL SA at 0 °C.

© 2020 by the authors. Submitted for possible open access publication under the terms and conditions of the Creative Commons Attribution (CC BY) license (http://creativecommons.org/licenses/by/4.0/).
